# Bifunctional scaffolds: strategic bridges coordinating osteosarcoma therapy and bone regeneration

**DOI:** 10.3389/fbioe.2026.1825326

**Published:** 2026-04-24

**Authors:** Nanjian Xu, Yang Xu, Weihu Ma, Fang Yang

**Affiliations:** 1 Spine Surgery Center, Ningbo No.6 Hospital, Ningbo City, Zhejiang, China; 2 Ningbo Clinical Research Center for Orthopedics, Sports Medicine and Rehabilitation, Ningbo, Zhejiang, China; 3 Department of Oncology, The Third Medical Center of Chinese PLA General Hospital, Beijing, China; 4 Department of Nursing, Ningbo No.6 Hospital, Ningbo City, Zhejiang, China

**Keywords:** bifunctional scaffolds, bone regeneration, localized drug delivery, osteosarcoma, stimuli-responsive materials

## Abstract

Osteosarcoma (OS), recognized as the most common primary malignant bone tumor, presents substantial clinical challenges. The current standard of care, involving extensive surgical resection followed by adjuvant chemotherapy, often leads to critical-size bone defects and is hampered by high risks of local recurrence, metastasis and systemic toxicity. Conventional bone scaffolds are constrained by their purely mechanical role, lacking the inherent bioactivity required for a therapeutic function within the regenerative microenvironment. This significant and unresolved clinical challenge has driven the advancement of sophisticated biomaterial platforms aimed at achieving the dual objectives of effective osteosarcoma elimination and concurrent bone tissue regeneration. This review comprehensively explores the design principles of these scaffolds, detailing their use as a structural base and the integration of key components for antineoplastic strategies and osteogenesis. Furthermore, it delves into advanced smart material systems, including stimuli-responsive drug release platforms and 3D printing technologies for creating patient-specific implants. The discussion also encompasses the critical *in vitro* and *in vivo* evaluation models used to assess the efficacy of these platforms. Finally, the review addresses the current challenges in balancing oncotherapy with regeneration and provides insightful perspectives on the future of this promising field, highlighting the potential of these engineered scaffolds to serve as strategic bridges connecting tumor ablation to functional bone reconstruction.

## 1 Introduction

Osteosarcoma (OS) is the most common and aggressive primary malignant tumor of the skeletal system, predominantly affecting adolescents and children, and poses a grave threat to patients’ quality of life and long-term survival (Yu and Yao, 2024; Beird et al., 2022; Chen et al., 2021). Although multidisciplinary treatment strategies developed over the past decades—including neoadjuvant chemotherapy, wide surgical resection, and individualized radiotherapy—have raised the 5 year survival rate to 60%–70%, prognosis deteriorates precipitously once distant metastasis or postoperative local recurrence occurs (Wu et al., 2023; Sun et al., 2024; Wang L. et al., 2024). Large bone defects are often caused by bone tumors invading and extensive resection, and have always been a challenging problem in orthopedic clinical practice (Lu et al., 2025; Ji et al., 2026; Li C. et al., 2024). The traditional strategy follows a stepwise approach of “first complete resection - auxiliary chemotherapy to clear residual lesions-second-stage functional reconstruction”(Donati et al., 2000; Alman et al., 1995), which not only has a lengthy treatment process but also leaves an inflammatory, hypoxic acidic pH, reactive oxygen species (ROS) and cell-suppressed local microenvironment due to the risk of tumor recurrence, making subsequent bone regeneration extremely difficult (Dang et al., 2020). These pathological features significantly compromise the efficacy of traditional bone grafts or inert scaffolds, which lack the capacity to modulate immune responses, counteract oxidative stress, or actively participate in tissue remodeling (Wang Y. et al., 2024). Therefore, developing a bifunctional scaffold that can continuously inhibit the residual/recurrent bone sarcoma and simultaneously induce efficient osteogenesis has become a key breakthrough to break the deadlock between “tumor control” and “bone defect repair” and actively orchestrate the regeneration process while maintaining oncological safety.

To address these challenges, bifunctional scaffolds have emerged as a promising strategy that integrates tumor-targeting therapy with bone regeneration in a single platform. These advanced constructs are designed not only to provide mechanical support but also to deliver antineoplastic agents, modulate immune responses, and create a pro-regenerative microenvironment (Long et al., 2021). By incorporating stimuli-responsive materials, bioactive ions, and controlled drug release systems, these scaffolds can adapt to the changing conditions of the defect site, ensuring that therapeutic molecules are released precisely when and where they are needed. Moreover, the advent of 3D printing and additive manufacturing has enabled the fabrication of patient-specific implants with tailored architectures, further enhancing the integration and performance of these bifunctional systems. This review aims to provide a comprehensive overview of the design rationale, key components, and evaluation strategies of bifunctional scaffolds, and to discuss the current challenges and future directions in translating these innovative platforms into clinical practice.

## 2 Design principles and key components of bifunctional scaffold

**FIGURE 1:**
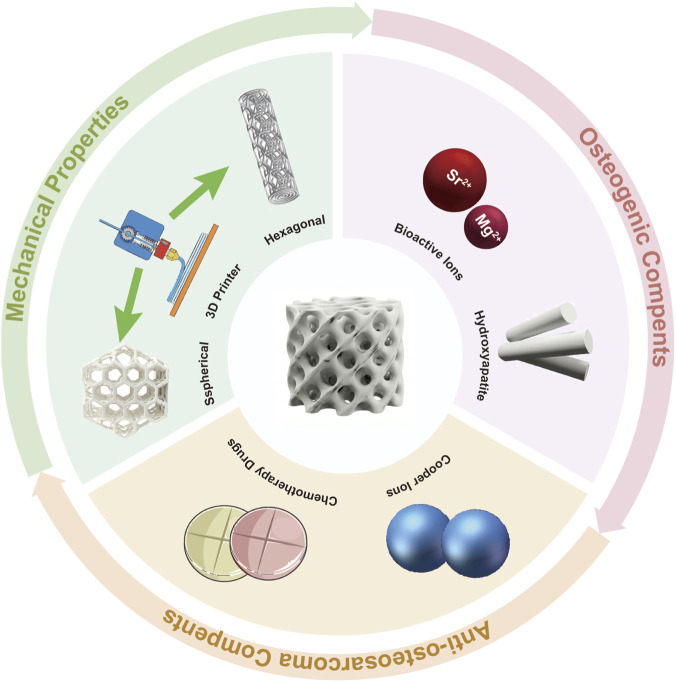
Key components of bifunctional scaffold.

This category of scaffold controls cell migration, fluid transport, mechanical stability, and the presentation of biochemical signals throughout the entire process of tumor eradication coupled with bone regeneration. Therefore, rational design of its structural foundation determines whether the effective components for anti-tumor or bone-promoting functions can be released at the appropriate dosage, duration, and location, while also withstanding the cyclical loads of the musculoskeletal system (Figure 1 inserted).

### 2.1 Scaffold-based mechanical strategies

The mechanical design of the dual-function bone tumor support must concurrently satisfy load-bearing criteria. It should facilitate localized delivery of anti-tumor agents while also establishing an optimal mechanical microenvironment conducive to bone regeneration. Triply periodic minimal surface (TPMS) architectures—particularly gyroid and diamond lattices—allow precise tuning of stiffness, strength and permeability by adjusting unit-cell size and porosity; gyroid scaffolds with pore sizes <800 µm achieve elastic moduli >6 GPa and compressive yield strengths >133 MPa, matching the lower bound of cortical bone and thereby avoiding stress shielding (Naghavi et al., 2022). Compared with diamond lattices, gyroids exhibit more homogeneous stress distribution, which delivers uniform mechanical cues to adherent cells and promotes balanced osteogenic signaling (Yang et al., 2019). These scaffolds’ design facilitates bone conduction and bone regeneration after bone tumor surgery. Incorporating bioactive ceramics (e.g., hydroxyapatite, biphasic calcium phosphate) or metallic nanofillers (e.g., copper ions, graphene) into the polymer matrix further raises compressive modulus and yield strength while providing sites for drug loading; Cao et al. indicates that introducing Cu^2+^ ions for cross-linking in the biphasic hydroxyapatite (BCP) matrix can significantly enhance the compressive modulus and shear strength of the scaffold. Cu^2+^ also possesses biological activities such as anti-tumor, antibacterial, and promoting angiogenesis, enabling the scaffold to provide mechanical support while inhibiting tumor recurrence (Cao et al., 2024). Multifunctional composites such as PEEK scaffolds coated with HA and graphene nanosheets combine high mechanical integrity with photothermal conversion, enabling on-demand laser-triggered drug release without compromising structural stability (Zhu et al., 2021). Multiple studies have confirmed that scaffolds loaded with anti-tumor drugs can simultaneously inhibit tumor growth and promote bone formation *in vivo*, demonstrating that a mechanically robust scaffold can act as a “bone-replacement-plus-pharmacy” platform while also providing a supportive matrix for the recruitment of host progenitor cells (Liang et al., 2024; Jing et al., 2023; Wei et al., 2021). 3D printing technology enables gradient modulus design, which allows scaffolds to match the host bone’s elastic modulus while mitigating stress shielding. Furthermore, by employing topological optimization and bionic design, it can mimic the mechanical properties of bone trabeculae, thereby enhancing the scaffold’s load-bearing capacity and fatigue resistance. This personalized adaptability to complex post-resection bone defects has made 3D printing a significant focus in osteosarcoma treatment research (Table 1 inserted).

**TABLE 1:** Recent examples of 3D-printed bifunctional scaffolds for anti-osteosarcoma.

*Biomaterials*	*Tumor therapy*	*Bone regeneration*	*Ref*.
3D-printed sandwich/MTO-CuHCF hydrogel scaffold	Chemical kinetics(Cu^2+^) + chemotherapy(MTO) + photothermal therapy(CuHCF)	Porous degradable structure of scaffold	Su et al. (2025)
3D-printed Se/Sr/Zn tri-element-doped HA/PCL scaffold	SeO_3_^2-^ induces ROS burst and mitochondrial apoptosis in osteosarcoma cells	The continuous release of Sr^2+^/Zn^2+^ induces osteogenesis	Huang et al. (2023)
3D-printed SrTiO_3_ nanoparticles/EGCG hybridized Sr bioglass scaffold	External ultrasound - piezoelectric ROS + EGCG demethylation” AND logic gate triggers PANoptosis	The continuous release of Sr^2+^ induces osteogenesis	Wang J. et al. (2025)
3D-printed Pt(IV)-PDA@Pt nanoparticles/PLLA bioglass scaffold	Chemotherapy - photothermal - immunotherapy for combined anti-osteosarcoma treatment	–OH of PDA/Ar–OH chelate Ca^2+^+ sustained-release Pt(II) micro-stimulation in combination with mild photothermal stress synergistically promotes osteogenesis	Yan et al. (2025)
3D printed Ti–6Al–4V scaffold + gelatin/sodium alginate hydrogel	Chemical kinetics(MgO_2_) + chemotherapy(Curcumin/Paclitaxel) + photothermal therapy(Y, Yb, Er)	The continuous release of Zn^2+^/Mg^2+^ induces osteogenesis	Ji et al. (2026)
3D printed bioceramic scaffold+2D metal-polyphenol network (MPN) nanosheets (EARu)	Chemical kinetics(Ru^2+^/Ru^3+^ induces ferroptosis)+ photothermal therapy(EARu)	EARu eliminates ROS and reduces inflammatory responses + Ca^2+^, Si^4+^, and P^5+^ induce osteogenesis	Jian et al. (2025)
3D printed titanium alloy scaffold	Chemotherapy(PH-responsive release of paclitaxel)	Porous structure of scaffold	Fan et al. (2023)
3D-printed CMB bioglass scaffold	Photothermal therapy(loaded cerium-doped molybdenum blue nanowires)+ROS	Synergistic effect of bone regeneration by Ce^3+^ with Ca^2+^, Si^4+^, and P^4+^ in Bioglass	Lu et al. (2024)
3D-printed GBS/CCP/PLA scaffold	Chemical kinetics(Cu^2+^)+ cuproptosis	Cu^2+^+nHA + porous structure of scaffold	Xie et al. (2025)
3D printed titanium alloy scaffold	Simvastatin induces ferroptosis	Simvastatin upregulates the expression of BMP-2	Jing et al. (2023)
3D-printed ICTO nanoparticle-coated HA scaffold	ICTO nanoparticles catalyze endogenous H_2_O_2_ to produce ROS	ICTO nanoparticles catalyze endogenous H_2_O_2_ to produce O_2_, alleviating hypoxia and inhibiting HIF-1α, while simultaneously enhancing the expression of osteogenic genes	Rong et al. (2025)
3D-printed MgO_2_/PLGA scaffold	MgO_2_ decomposes to release H_2_O_2_	The continuous release of Mg^2+^ induces osteogenesis	Li L. et al. (2024)

### 2.2 Scaffold-based drug delivery strategies

In the treatment of osteosarcoma using conventional drug delivery methods, the comparatively reduced vascularization of bone tissue relative to soft tissues constrains the efficiency of drug transport to the targeted site. Consequently, clinical practice frequently necessitates the use of systemic administration to offset the inadequate local perfusion (Foroughi et al., 2016; Bordone and Bettencourt, 2023). Consequently, the objective for this category of medication has consistently been to establish a delivery method that is long-period, sustained, and precise (Paolini et al., 2019). To this end, bone scaffolds implanted directly into the resection cavity are emerging as a promising “bone-replacement-plus-pharmacy” platform (Mao et al., 2024). Owing to their architectural conformity to the defect, these constructs can (i) bypass the scarce cortical blood supply and place the drug within micrometres of residual tumor nodules (Liang et al., 2024), (ii) provide a high, yet tunable, local effective concentration while keeping plasma levels low (Wang C. et al., 2022), and (iii) release chemotherapeutics in a sustained fashion that matches the relatively slow bone-healing time-scale (Zhou et al., 2017). Moreover, the scaffold surface can be functionalised with tumor-targeting ligands or stimuli-responsive coatings, enabling on-demand pulsatile or photo-activated drug bursts precisely where malignant cells persist (Rong et al., 2025). Simultaneously, the same scaffold serves as an osteoconductive and osteoinductive matrix, supporting the recruitment and differentiation of host progenitor cells, thereby coupling eradication of osteosarcoma with segmental bone regeneration in a single surgical step (Zhang et al., 2024).

The anti-osteosarcoma components usually possess certain cytotoxicity, which not only kills tumor cells but also causes damage to local or systemic normal tissues (Maleki et al., 2021; Liang et al., 2024). Hence, in the development of a bifunctional scaffold for osteosarcoma treatment, it is imperative to achieve a balance between effective anti-tumor properties and the promotion of osteogenic activity, while concurrently minimizing local cytotoxic effects to the greatest extent feasible (Gu et al., 2013). The development of these localized delivery systems is fundamentally based on the deliberate integration of therapeutic agents either within the scaffold matrix or on its surface. Common strategies include physical adsorption, covalent conjugation, or encapsulation within micro/nanocarriers that are subsequently integrated into the scaffold architecture (Liang et al., 2024). For instance, doxorubicin can be loaded into the pores of bioceramics (e.g., hydroxyapatite) (Liu et al., 2021; Wang Y. et al., 2024)or bound to natural polymer coatings like chitosan (Ta et al., 2009; Amiryaghoubi et al., 2022), allowing for diffusion-controlled release. More advanced approaches employ co-delivery systems to address the multifaceted challenge. Scaffolds can be engineered to sequentially release an initial burst of chemotherapeutic agent to eliminate residual tumor cells, followed by a sustained release of osteogenic ions (e.g., Sr^2+^, Mg^2+^) to initiate and support bone regeneration (Wang W. et al., 2025; Chu et al., 2025). The timing of this treatment regimen is essential to mitigate the suppressive impact of high-dose cytotoxic agents on the migration and differentiation of mesenchymal stem cells and osteoblasts, thereby creating a permissive window for bone regeneration after the primary tumoricidal action.

### 2.3 Anti-osteosarcoma components within the bifunctional scaffold

**FIGURE 2:**
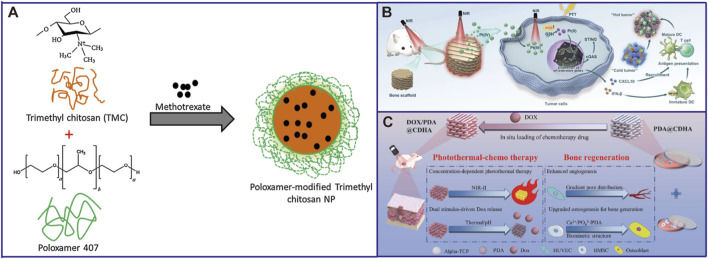
Different anti-osteosarcoma components in the scaffolds. **(A)** Methotrexate (Li et al., 2017); **(B)** Platinum (Yan et al., 2025); **(C)** Doxorubicin (Wang Y. et al., 2024).

Scaffold-based drug delivery systems embed chemotherapeutic agents (such as cisplatin, doxorubicin, methotrexate) into porous, biocompatible scaffold materials, enabling high-local-concentration drug release while markedly reducing systemic toxicity (Yang et al., 2020a; Chaudhari et al., 2024; Wang M. L. et al., 2022) (Figure 2 inserted).

Among the anti-osteosarcomaa drugs, platinum-based drugs stand out particularly. Yan et al. designed a low-toxicity Pt(IV) prodrug through oxidation of cisplatin. This prodrug can respondively release highly cytotoxic Pt(II) under the condition of high glutathione (GSH) in the tumor microenvironment, thereby inducing DNA damage and activating the cGAS-STING immune pathway, converting “cold tumors” into “hot tumors”. This scaffold, under near-infrared light irradiation, not only enhances the sensitivity of tumor cells to chemotherapy through photothermal effect, but also accelerates the on-demand release of Pt(II) through local heating, achieving precise and synergistic anti-tumor effects (Yan et al., 2025).

Apart from platinum-based drugs, doxorubicin (DOX), as another type of broad-spectrum chemotherapy drug, also plays a central role in the multifunctional scaffolds. One strategy is to use polydopamine-functionalized calcium-deficient hydroxyapatite (CDHA) 3D-printed scaffolds to load DOX. This scaffold also has photothermal conversion capabilities and can achieve continuous release of DOX under near-infrared light triggering, thereby synergistically efficiently inhibiting osteosarcoma through both photothermal and chemotherapy approaches (Wang L. et al., 2024). Another innovative design is based on the unique layered structure of magnesium silicate (MgSiO_3_) fiber membrane scaffolds, which allows for the migration and substitution of cations (such as Mg^2+^). The research first utilized the high affinity of negatively charged indocyanine green (ICG) to the scaffolds to achieve a high drug loading capacity. Then, through the electrostatic attraction between ICG and positively charged doxorubicin (DOX), a large number of DOX molecules were further adsorbed. The photothermal effect could disrupt the electrostatic interaction between ICG and DOX, thereby jointly triggering the targeted release of DOX and achieving the purpose of synergistic enhancement (Mao et al., 2024).

Methotrexate (MTX), as a classic antimetabolite drug, inhibits the synthesis of DNA in tumor cells by blocking dihydrofolate reductase. However, its clinical application is limited by its poor water solubility and systemic toxicity (Nie et al., 2025). To overcome this limitation, the methotrexate-loaded pluronic-chitosan nanoparticles (MTCN) can be efficiently internalized by cells, significantly enhancing the accumulation of MTX within osteosarcoma cells. This results in a stronger cytotoxicity and apoptosis-inducing ability compared to free drugs, and they possess pH-responsive release properties. They release more rapidly in the acidic tumor microenvironment, facilitating enrichment at the tumor site and reducing systemic side effects (Li et al., 2017). Another advanced strategy is to synergistically deliver chemotherapy drugs and immunostimulants, constructing “chemotherapy-sensitized *in situ* vaccines”. Wang et al. utilized bovine serum albumin (BSA) as a scaffold and synthesized calcium phosphonate nanoparticles (CpG-MTX@BSA-CaZol) through biomineralization. This system carried MTX, immunomodulators CpG ODNs, and a bone-targeting agent zoledronic acid (Zol) (Wang D. et al., 2023). Therefore, the core of modern anti-osteosarcoma scaffolds or carriers has shifted to how to intelligently utilize chemotherapy drugs, releasing them precisely and spatially to maximize the synergistic effect of direct killing and immune activation.

### 2.4 Osteogenic components within the bifunctional scaffold

While eliminating residual tumor cells is the primary therapeutic goal, the ultimate success of a bifunctional scaffold hinges on its ability to effectively regenerate functional bone tissue within the defect site (Dong H. et al., 2025; Zhang et al., 2024). To achieve this, scaffolds are engineered with a range of osteogenic components designed to recruit host progenitor cells, promote their osteogenic differentiation, and guide the formation of mineralized bone matrix.

Wang et al. incorporated SrTiO_3_ nanoparticles into 3D-printed scaffolds, significantly enhancing their piezoelectric properties, thereby improving the efficiency of reactive oxygen species (ROS) generation under ultrasonic triggering. Moreover, the strontium ions released by SrTiO_3_ itself possess biological activity that promotes osteogenic differentiation and bone regeneration, endowing the scaffolds with dual functions of anti-tumor and accelerating bone defect repair (Wang et al., 2025; Liu et al., 2023). The bioactive nano-composite hydrogel developed by Chu et al. can not only kill osteosarcoma but also continuously release magnesium ions. The magnesium ions drive the self-assembly of the hydrogel through dynamic coordination, endowing it with injectability and stress relaxation properties. Moreover, the continuously released Mg^2+^ significantly promotes the osteogenic differentiation of BMSCs and the repair of bone defects (Chu et al., 2025; Yu et al., 2023).

In addition, hydroxyapatite (HA), a common inorganic substance in bone tissue engineering, is also widely used in bifunctional scaffolds for osteosarcoma. Incorporating HA into the scaffold can significantly improve the hydrophilicity of the biomaterial surface, making it more conducive to cell adhesion and proliferation. Moreover, the continuous release of calcium ions and phosphate ions in HA provides a favorable microenvironment for bone regeneration (Xie et al., 2025). Fang et al. used black phosphorus as a piezoelectric nanomaterial. Under ultrasonic stimulation, it produced ROS and, in combination with NO gas, achieved efficient tumor killing. The degradation product, phosphate ion, is non-toxic and also has osteogenic and biomineralization functions, facilitating subsequent bone regeneration (Fang et al., 2025). In the harsh microenvironment of osteosarcoma treatment, inorganic compound ions, due to their outstanding stability and controlled release properties, can effectively resist degradation and continue to exert their effects. The core advantage lies in their ability to both promote osteogenesis and directly inhibit tumors or regulate immunity, achieving efficient synergy between anti-tumor and regeneration. In contrast, traditional bioactive molecules are prone to inactivation, have high costs, and have limited functions in this environment. Inorganic ions, as natural components of bone tissue, provide a more direct, economical, and integrated solution (Gu et al., 2023; Wang Y. et al., 2023).

## 3 Advanced Smart/Responsive scaffolds

**FIGURE 3:**
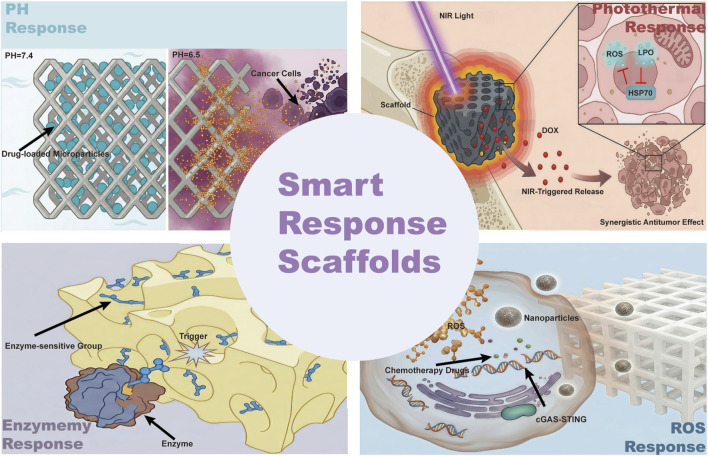
Catelogories of smart response scaffolds.

The integration of “therapy” and “regeneration” functions into a single scaffold hinges on the premise that drugs, ions, or gene signals must be activated or terminated at the correct time, dosage, and spatial site. To this end, researchers have recently introduced the concept of stimuli-responsiveness into the bone tumor-bone defect microenvironment, achieving on-demand on/off, pulsatile, or self-accelerating release by sensing endogenous or exogenous signals such as pH, ROS, enzymes, magnetic fields, light, and ultrasound. The design principles, triggering mechanisms, and application progress of these systems in post-operative osteosarcoma scenarios are reviewed below according to four categories (Figure 3 inserted).

### 3.1 pH-response

pH-responsive functional materials can undergo physical or chemical property changes in response to variations in environmental pH values, such as swelling, shrinking, dissolving, or releasing loaded substances (Kanamala et al., 2016). In the microenvironment of bone tumors, due to the abnormal metabolism of tumor cells, the local pH value is usually lower than that of normal tissues, presenting an acidic state (Yang et al., 2020b; Avnet et al., 2021; Zhu et al., 2023). During bone regeneration, the pH value in the bone defect area also undergoes certain changes, such as a decrease in the initial stage of the inflammatory response or microenviroment of mature osteoclast, and gradually returns to normal as the osteogenesis process progresses (Dou et al., 2021).

By taking advantage of this pH value difference, pH-responsive functional materials that can specifically respond can be designed to achieve precise killing of bone tumor cells or precise regulation to promote bone regeneration (Shi et al., 2022). Zhu et al. developed a hydrogel capable of self-assembling under neutral conditions for efficient drug encapsulation. This system exhibits pH-responsive behavior, enabling intelligent and sustained doxorubicin release in acidic tumor-mimicking microenvironments (pH 5.8), with a cumulative drug release 3.6-fold higher than that observed at physiological pH 7.4 (Zhu et al., 2023). Fan et al. developed a composite implant system integrating a 3D-printed titanium alloy scaffold with a pH-responsive polyethylene glycol-paclitaxel prodrug. This construct remains stable under physiological conditions (pH 7.4) to facilitate bone repair, while rapidly responding to the weakly acidic tumor microenvironment (pH 6.5) to trigger localized paclitaxel release, thereby achieving dual functionality in bone defect regeneration and local suppression of osteosarcoma recurrence (Fan et al., 2023).

### 3.2 ROS-response

Reactive oxygen species (ROS) are key signaling molecules in both the tumor microenvironment and the bone repair microenvironment (Weinberg et al., 2019; Sun et al., 2023). They play a bidirectional regulatory role in the proliferation of bone tumor cells and the differentiation of bone regeneration cells: The level of ROS in the bone tumor microenvironment is significantly elevated, which maintains the malignant phenotype of the tumor by activating downstream oncogenic signaling pathways; while the abnormal accumulation of ROS during bone injury repair inhibits the function of osteoblasts and hinders angiogenesis, leading to delayed bone healing (Ye et al., 2024; Tornín et al., 2021; Lee et al., 2022). Based on this, ROS-responsive scaffolds, with the core advantage of “precisely regulating the level of ROS in the microenvironment”, achieve the synergistic regulation of bone tumor-targeted treatment and bone regeneration, and have become a research hotspot in the field of bone tissue engineering (Ding et al., 2026). Wu et al. developed a nanoparticle (NP3) that responds to ROS. This nanoparticle is specifically triggered for degradation within tumor cells in the high-ROS environment. Upon degradation, the nanoparticle releases cisplatin and curcumin, which synergistically induce DNA damage, leading to the activation of the cyclic GMP-AMP synthase-stimulator of interferon genes (cGAS-STING) signaling pathway. Simultaneously, the released manganese ions act as an immune adjuvant to further enhance this pathway, ultimately promoting the maturation of dendritic cells and the infiltration of CD8^+^ T cells, thereby reshaping the tumor immune microenvironment (Wu et al., 2025).

### 3.3 Enzyme-response

The design of enzyme-responsive functional materials is based on the specific interaction between enzymes and their substrates. In the bone tumor microenvironment, the activity and concentration of enzymes secreted by tumor cells (such as matrix metalloproteinases MMPs and cathepsins) are significantly higher than those in normal tissues (Li et al., 2020; Zhou et al., 2019; Ji et al., 2013). During bone regeneration, enzymes secreted by osteoblasts and osteoclasts (such as alkaline phosphatase ALP and acid phosphatase ACP) also participate in the remodeling of bone tissue (Kaplan, 1972; Liu et al., 2025). By introducing the substrates of enzymes or enzyme-sensitive groups into the material structure, when the material is exposed to a specific enzyme environment, the enzyme will catalyze the hydrolysis or transformation of the substrate, thereby triggering the degradation of the material, the release of drugs, or the activation of bioactive molecules. This enzyme-responsive mechanism provides the possibility for targeted treatment of bone tumors and precise regulation of bone regeneration (Wang et al., 2025). Wang et al. developed a 3D-printed bioactive glass scaffold integrated with a single-atom iron catalyst (FeSAC), which, through its Fenton catalytic activity, selectively generates hydroxyl radicals in the osteosarcoma microenvironment to destroy the tumor. This engineered scaffold also utilizes the local photothermal effect to enhance its nanozyme catalytic therapeutic function, achieving a synergistic effect of antibacterial and promoting bone regeneration (Wang et al., 2021).

### 3.4 Photothermal-Response

Photothermal therapy, as an emerging tumor treatment modality, demonstrates significant potential in the comprehensive treatment of osteosarcoma due to its controllable time and space, minimally invasive nature, and high precision. Especially when combined with multifunctional biomaterial scaffolds, it achieves synergistic enhancement of tumor ablation and bone regeneration (Pan et al., 2019; Yin et al., 2021; Tan et al., 2021).

Traditional photothermal therapy usually requires a temperature higher than 50 °C to completely kill tumor cells, but this inevitably damages the surrounding normal tissues. To solve this problem, researchers turned to mild photothermal therapy (Mild PTT) (Hu et al., 2024; Zeng et al., 2022), which uses a temperature lower than 48 °C to induce the death of tumor cells (Yan et al., 2024). However, the effect of mild photothermal therapy is limited because the damage it causes can be repaired by tumor cells through stress-induced heat shock proteins (HSPs), resulting in heat tolerance. To overcome this obstacle, cutting-edge research combines photothermal therapy with ferroptosis. During ferroptosis, the LPO and ROS produced can effectively inhibit the expression of HSP70, thereby eliminating the resistance of tumor cells to mild photothermal therapy and achieving a synergistic and effective anti-tumor effect (Yan et al., 2024).

**FIGURE 4:**
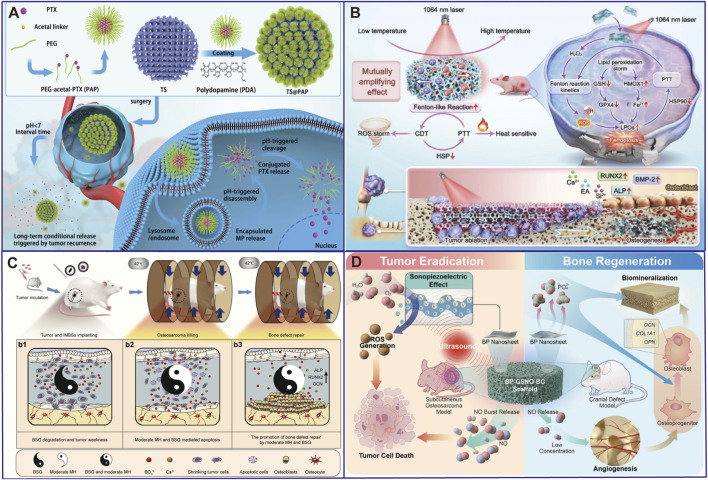
Advanced Smart/Responsive scaffolds. **(A)** pH-response scaffold (Fan et al., 2023); **(B)** Photothermal-Response bifunctional scaffold (Jian et al., 2025); **(C)** Magnetothermal-Response bifunctional scaffold (Fan et al., 2025); **(D)** Microwave response bifunctional scaffold (Fang et al., 2025).

In terms of material design, in order to achieve deeper tissue penetration and higher treatment safety, the research focus is shifting from the first near-infrared window (NIR-I, 650–950 nm) to the second near-infrared window (NIR-II, 1,000–1,350 nm). NIR-II light has lower tissue self-heating, a higher maximum allowable exposure dose, and deeper penetration depth, and thus has higher value in clinical applications (Wang et al., 2019; He et al., 2021). In addition to photothermal ablation, the multifunctional stent is also designed to work in conjunction with chemotherapy. For instance, a dopamine-functionalized calcium-deficient hydroxyapatite 3D-printed stent can load doxorubicin (DOX) and achieve near-infrared light-triggered drug release (Wang Y. et al., 2024). Under NIR-II laser irradiation, the photothermal effect generated by the Egyptian blue nanosheet-decorated scaffold synergizes with the bioactive ion release (Ca, Cu, Si) to achieve concurrent photothermal ablation of osteosarcoma and enhanced osteogenesis, thereby realizing the bifunctional therapeutic outcome of tumor elimination and bone regeneration (He et al., 2021) (Figure 4; Table 2 inserted).

**TABLE 2:** Examples of stimuli-responsive drug release delivery system used for osteosarcoma therapy.

*Responsive Types*	*Responsive threshold*	*Biomaterials*	*Released drugs*	*Tested models*	*Ref*.
pH-response	pH 5.8 reduction release	Self-assembling peptide P1	Doxorubicin	*In vitro*: Mouse K12 osteosarcoma cell line; in vivo: Subcutaneous inoculation	Zhu et al. (2023)
	pH ≤ 6.5 release	TiO_2_ nanotube arrays modified with polydopamine	Doxorubicin	*In vitro*: MG63 osteosarcoma cell line; in vivo:none	Xiao et al. (2024)
	pH ≤ 6.5 release	3D printed Ti–6Al–4V scaffold modified with polydopamine	Paclitaxel	*In vitro*: 143B,MG63,HOS,Saos-2,U2OS; in vivo: subcutaneous inoculation	Fan et al. (2023)
	pH ≤ 6.5 release	Surface phosphorylation-modified poly(HEMA-co-MMA)	Doxorubicin	*In vitro*:MG63,HOS; in vivo: none	Sreeja et al. (2021)
	pH 6.2 release	Magnesium scaffold linked by a zirconium bond	Doxorubicin	*In vitro*:MG63; in vivo: subcutaneous inoculation	Ding et al. (2026)
ROS-response	10 mM H_2_O_2_	ROS-responsive polymer P1 + C8-Cis(IV) + curcumin - manganese ion complex	Cisplatin, Curcumin,Mn^2+^	*In vitro*:K7M2,HOS,143B,MG63; in vivo: Intramedullary bone marrow cavity inoculation of the tibia	Wu et al. (2025)
	100 μM H_2_O_2_	IL-11 engineered macrophage membrane + ROS-responsive amphiphilic molecule PCM	Doxorubicin	*In vitro*:143B,MG63,Saos-2,HOS,SJSA1,LM7; in vivo: subcutaneous inoculation + *in situ* inoculation of the femur	Jaing et al. (2024)
	5 mM H_2_O_2_	ROS-responsive benzeneboronic acid polymer RP4	Saporin	*In vitro*: 143B; in vivo: Subcutaneous inoculation	Lv et al. (2023)
	0.1% H_2_O_2_(w/v)	Magnesium scaffold linked by a zirconium bond	Doxorubicin	*In vitro*: MG63; in vivo: subcutaneous inoculation	Ding et al. (2026)
Enzyme-response	ALP response	Mesoporous bioactive glass	Doxorubicin	*In vitro*: HOS; in vivo: none	Polo et al. (2017)
	MMP9 response	Carbon quantum dots	Methotrexate	*In vitro*: 143B,HOS; in vivo: subcutaneous inoculation	Fu et al. (2025)
Photothermal-response	808 nm NIR-II (1 W/cm^2^)	Polydopamine (PDA) Calcium-deficient hydroxyapatite	Doxorubicin	*In vitro*: MG63,HOS; in vivo: subcutaneous inoculation	Wang L. et al. (2024)
	1,064 nm NIR-II (0.2–1.5 W/cm^2^)	Egyptian blue nanosheets CaCO_3_-PCL CaPCu	None	*In vitro*: 143B,HOS; in vivo: subcutaneous inoculation	He et al. (2021)
	1,064 nm NIR-II (0.25–2 W/cm^2^)	Ce_10_Mo_90_O_350_·4H_2_O Bioglass	None	*In vitro*: MG63,LM8; in vivo: subcutaneous inoculation+ *in situ* inoculation	Lu et al. (2024)
	1,064 nm NIR-II (0.25–1.0 W/cm^2^)	Gallogen + Ru^3+^/Ru^2+^(EARu nanoparticles); Bioglass	None	*In vitro*:143B; in vivo: subcutaneous inoculation	Jian et al. (2025)
	808 nm NIR-II (1 W/cm^2^)	Polydopamine (PDA); PCL; nHA; MgO_2_	None	*In vitro*:143B; in vivo: subcutaneous inoculation	Haixia et al. (2024)
Magnetothermal-response	AMF:550 kHz 15–20 a	Zn_0_._54_Co_0_._46_Cr_0_._6_Fe_1_._4_O_4_(MNP) Borosilicate bioactive glass Chitosan-based self-setting adhesive	None	*In vitro*:143B; in vivo: subcutaneous inoculation+ *in situ* inoculation of the tibia	Fan et al. (2025)
	AMF: 560 kHz 7–8 a	Fe_3_S_4_ Ca_2_MgSi_2_O_7_	None	*In vitro*:MG63; in vivo: subcutaneous inoculation	Zhuang et al. (2021)
	AMF: 305 kHz 5 kW	Fe_3_O_4_ PLMC; m-Hap Microporous SiO_2_ stabilizer	None	*In vitro*: UMR-106 *In vivo*: subcutaneous inoculation	Wang et al. (2025)
Ultrasound-response	UF:1 MHz 1.2 W cm^-2^	Black phosphorus nanosheets S-Nitrosoglutathione Bioglass	None	*In vitro*:MG63; in vivo: subcutaneous inoculation	Fang et al. (2025)
	UF:1 MHz 1.2 W cm^-2^	SrTiO_3_ nanocrystals Bioglass	None	*In vitro*:HOS; in vivo: subcutaneous inoculation	Wang et al. (2025)
Microwave response	MWF:433 MHz 1.8 W	Zeolite imidazolate framework-8(ZIF-8) Ti6Al4V	Doxorubicin	*In vitro*:K7M2; in vivo: *in situ* inoculation of the femur	Ma et al. (2023)

## 4 *In Vitro* evaluation models

Osteosarcoma (OS), as a highly heterogeneous malignant tumor, presents a dual challenge of bone defect repair and tumor recurrence risk after treatment, posing a severe challenge to preclinical evaluation models. Traditional two-dimensional (2D) cell culture systems, due to their inability to simulate the complexity of the *in vivo* tumor microenvironment (TME), including cell-extracellular matrix (ECM) interactions, biochemical gradients, and biomechanical signals, result in a significant gap between drug screening results and clinical translation. To overcome these limitations, the scientific community has shifted its focus to more physiologically relevant three-dimensional (3D) *in vitro* models and their corresponding *in vivo* validation systems.

In terms of *in vitro* model construction, 3D culture systems based on scaffolds have demonstrated great potential. These systems utilize biomaterials to provide physical support and biochemical cues for cell growth, thereby more realistically simulating the ecological niche of bone tumors. For instance, a study utilized methylacrylated platelet lysate (PLMA) hydrogel to encapsulate osteosarcoma cell spheres, successfully constructing a humanized 3D triple-culture model. This model not only supported tumor invasion but also replicated the synergistic interactions between tumor cells and stromal cells (such as osteoblasts and mesenchymal stem cells), and exhibited stronger drug resistance compared to non-scaffold spherical bodies (Monteiro et al., 2021). A study compared four different biomaterials (GelMA, Gel μRB, Collagen I, PLGA) combined with hydroxyapatite (nHA) as scaffolds. It was found that the choice of scaffold material significantly affects the phenotype and drug response of osteosarcoma cells. For instance, the GelMA + nHA scaffold exhibited the highest water absorption rate. Moreover, there were significant differences in the proliferation, expression of invasion-related genes, and sensitivity to chemotherapy drugs such as methotrexate and doxorubicin among osteosarcoma cells on different scaffolds (Monette et al., 2025). Wang et al. proposed an *in vitro* model of osteosarcoma based on a 3D-printed PLLA scaffold. By simulating the mechanical and structural characteristics of cortical bone and combining dopamine surface modification, it significantly enhanced the biological relevance of tumor cells in aspects such as cell morphology, energy metabolism, extracellular matrix expression, and signal pathway activation. This model is more similar to the patient’s tissue in the expression of multiple clinical biomarkers and provides a more physiologically representative three-dimensional platform for the study of osteosarcoma mechanisms and drug screening (Wang C. et al., 2022).

Another systematic review comprehensively examined the scaffold-based 3D models used for drug screening in osteosarcoma, highlighting the advantages and challenges of different platforms such as hydrogels, macroporous scaffolds, decellularized matrix scaffolds, and microfluidic chips (osteosarcoma chips) in simulating tumor invasion, metastasis, and drug resistance. Among them, hydrogels are widely used due to their high water content and adjustable mechanical properties, while decellularized bone matrix scaffolds are better at maintaining the signal transduction and drug resistance phenotype of tumor cells because they retain the biochemical components of the natural ECM (Mandeda et al., 2025). Furthermore, microfluidic technology, by integrating dynamic perfusion, can simulate the vascularized environment in the body, providing a platform that is closer to physiological conditions for studying tumor-matrix interactions and drug metabolism (Mandeda et al., 2025).

Menshikh et al. used 3D-printed β-tricalcium phosphate (β-TCP) scaffolds as a bone simulation environment, and constructed an engineered osteosarcoma model. This model successfully replicated the key aspects of the tumor microenvironment by evaluating the compatibility of the scaffolds with osteosarcoma cell spheres, endothelial cells, and primary bone marrow mesenchymal stem cells, combined with their physical and chemical properties, and enhanced metabolic activity through dynamic perfusion in a triple culture system. The application of this model in the Adriamycin cytotoxicity test demonstrated that the triple culture system exhibited a significantly different drug accumulation pattern compared to the spherical cell monolayer culture, thereby proving its potential as a physiologically relevant preclinical platform (Menshikh et al., 2025).

In terms of simulating the ecological niche of tumor stem cells (CSCs), the researchers cultivated enriched osteosarcoma stem cells on a biomimetic scaffold to construct a 3D engineering model that could replicate the complexity of tumors in the body. Through continuous passage culture of tumor spheroids, the researchers enriched CSCs and optimized the inoculation strategy in a magnesium-doped hydroxyapatite (MgHA/CoII) composite scaffold. The final constructed model not only exhibited tumor-like characteristics *in vitro* but also demonstrated its tumorigenic potential in a chicken embryo chorionic allantoic membrane (CAM) *in vivo* model. This work lays the foundation for the future development of more complex systems that can simulate specific malignant behaviors such as metastasis and drug resistance (Bassi et al., 2024).

## 5 Current challenges and future perspectives

Despite significant advances in the incorporation of biochemical signaling molecules into bifunctional osteosarcoma scaffolds, several challenges persist in translating these engineered systems into clinical practice. One major hurdle lies in achieving spatiotemporal control over the release of osteogenic cues in a pathologically dynamic microenvironment. Tumor resection sites are often characterized by fluctuating pH, reactive oxygen species (ROS), and inflammatory cytokines (Sreeja et al., 2021; Li et al., 2025; Dong Y. et al., 2025), which can unpredictably degrade or prematurely activate signaling molecules such as BMPs, Wnt agonists, or growth factors. This compromises the intended osteogenic cascade and may inadvertently promote residual tumor progression if oncogenic pathways are co-stimulated (Zhang et al., 2024).

Another critical issue is the molecular crosstalk between osteogenesis and tumorigenesis. While BMP-2 and Wnt/β-catenin signaling are potent drivers of osteoblast differentiation, aberrant activation of these same pathways has been implicated in osteosarcoma aggressiveness and metastasis (Luo et al., 2008). This dual role necessitates a more nuanced understanding of how scaffold-delivered biochemical signals are interpreted by both normal progenitor cells and residual tumor clones. Future scaffold designs must incorporate cell-type-specific targeting strategies—such as aptamer-functionalized nanoparticles or promoter-activated gene delivery—to ensure that osteogenic signals are preferentially received by healthy host cells while sparing or even suppressing neoplastic populations (Zhang et al., 2024).

Furthermore, standardization of *in vitro* and *in vivo* models remains a bottleneck. Most current studies rely on monoculture systems or simplified 3D models that fail to recapitulate the heterogeneity of the post-resection tumor bed. There is a pressing need for patient-derived xenograft (PDX) models and humanized microphysiological systems that integrate immune components, ECM heterogeneity, and vascularization to more accurately assess the safety and efficacy of scaffold-delivered biochemical signals.

Looking forward, next-generation scaffolds must embrace multi-modal bio-responsive systems that integrate biochemical signaling with real-time feedback mechanisms. For instance, biosensors embedded within the scaffold could detect local VEGF or BMP-2 levels and modulate release accordingly. Additionally, CRISPR-based gene-activating scaffolds that transiently upregulate osteogenic transcription factors (e.g., Runx2, Osterix) in host progenitor cells—without transfecting tumor cells—represent a promising frontier. Finally, machine learning-assisted scaffold design, trained on multi-omics datasets from osteosarcoma patients, could predict optimal signaling molecule combinations and release kinetics tailored to individual tumor microenvironments.

In conclusion, while biochemical signaling molecules have transformed osteosarcoma scaffolds from passive supports into bio-instructive platforms, their clinical translation hinges on resolving the paradoxical interplay between regeneration and recurrence. Future efforts must prioritize precision delivery, cell-specific targeting, and microenvironmental adaptability to fully realize the potential of these intelligent constructs in post-oncological bone reconstruction.
